# Intrathoracic giant pleural lipoma: case report and review of the literature

**DOI:** 10.1186/1749-8090-8-196

**Published:** 2013-10-11

**Authors:** Ming Chen, Jun Yang, Lei Zhu, Heng Zhao

**Affiliations:** 1 Department of Thoracic Surgery, Shanghai Chest Hospital affiliated to Shanghai Jiao Tong University, Shanghai, China; 2 Department of pathology Surgery, Shanghai Chest Hospital affiliated to Shanghai Jiao Tong University, Shanghai, China

**Keywords:** Intrathoracic tumor, Pleural lipoma, Imaging, Right thoracotomy surgery

## Abstract

This report describes a giant pleural lipoma that arose from the pleura of the 7th anterior intercostal space and occupied approximately 75% of the right pleural cavity in a 49-year-old woman. The tumor was completely excised by right thoracotomy. The complete histopathological investigation showed pleural lipoma, and we made a review of literature.

## Background

Lipoma is mostly found within the subcutaneous areas of the body. Pleural lipoma is rare, which is usually located at the mediastinal, bronchial and pulmonary levels. The finding of a lipoma in the parietal pleura intrathoracic has been sporadically reported in the literature [1].

Most patients remain asymptomatic and the lipomas are incidentally found in a chest radiograph or a computed tomography (CT) examination.

Although pleural lipoma is benign tumor, it should be completely resected from diagnostic and therapeutic perspectives [2].

Here we report a case of pleural lipoma successfully resected through right thoracotomy with a review of the literature.

## Case presentation

A 49-year-old Chinese rural woman was admitted to our hospital with chest tightness for 10 days, complaining of mild shortness of breath without past medical history. She was a nonsmoker, and maintained a healthy weight of 50 kg for her 163 cm height. On examination, the patient was found to have absent breath sounds accompanied by dullness on percussion in the right low lung field. Laboratory workup was normal. The results of lung function tests, which showed FEV1/FVC was 99%, furthermore FVC was 64.1% of its predicted value, were consistent with the impression of restrictive ventilatory disorder result from thoracic large tumor.

**Figure 1:**
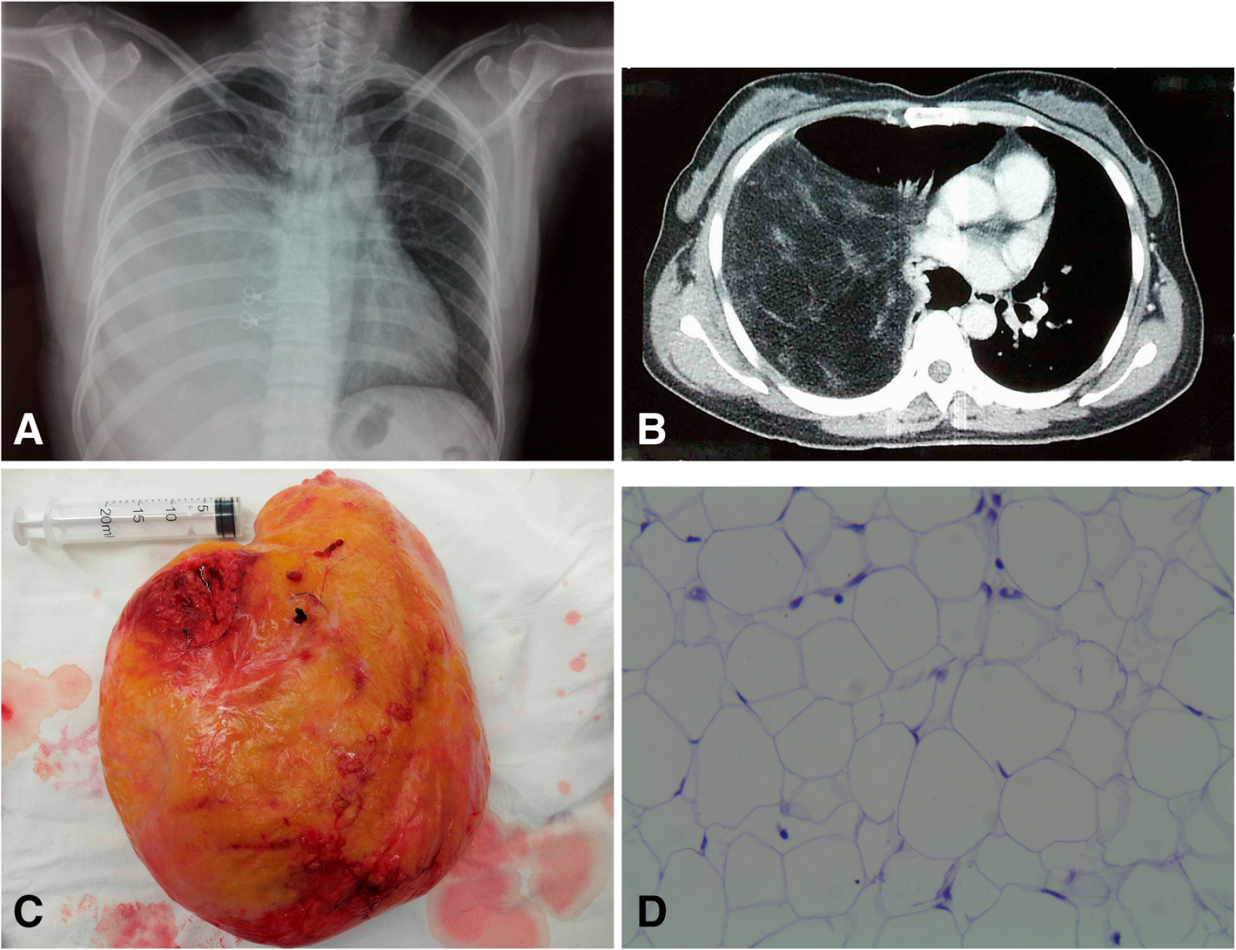
**A) chest X-ray, P-A view: shows huge mass in the right thoracic cavity. B)** A CT scan of the chest: shows a large fatty density with scattered stripped. Soft tissue density mass **C)** Macroscopic appearance of the intrathoracic lipoma **D)** Histological examination consistent with pleural lipoma (H&E,×200).

A routine chest roentgenogram (Figure 1A) showed a large homogenous mass in the lower half of the right lung field. A computed tomography (CT) scan of the chest revealed a large fatty density (-105 Hounsfield units) with scattered stripped soft tissue density (42 Hounsfield units) mass which compressed the right lower lobe (Figure 1B). A provisional diagnosis of intrathoracic lipoma was made.

Right thoracotomy revealed a round, yellowish, consistently soft tumor which had a smooth surface (Figure 1C), and occupied approximately 75% of the right pleural cavity. The tumor attached to the pleura of the 7th anterior intercostal space with its pedicle of 2.0 cm in diameter, compressing the right lower lobe of the lung. There was no adhesion between the tumor and the surrounding organs, and the entire mass was successfully extirpated en-bloc after ligation of its pedicle. The tumor weighed a total of 2850 grams and measured 25 × 20 × 18 centimeters.

Final histologic result was pleural lipoma. The investigation of this tumor revealed an encapsulated tumor with abundant mature adipose tissue, a few scattered strands of fibro connective tissue, no presence of sarcomatous changes (Figure 1D).

The patient’s postoperative course was uneventful, with no recurrence on one-year follow-up.

## Discussion

Lipoma is a benign mesenchymatous tumour. Clinically, a large number of lipomas are found within the subcutaneous areas of the body, but intrathoracic lipomas are rare and they can be located at the mediastinal, bronchial and pulmonary levels. A pleural lipoma is extremely rare [3,4].

Tateishi et al. [5] reported intrathoracic lipomas were relevant to obesity. However, a retrospective study conducted by Sakurai et al. [6] showed that only three out of the ten patients were described as obese (body mass index [BMI] > 25 kg/m2).In current case the patient was thin with the BMI of just 20.3 kg/m2. Clinically, most lipomas become apparent in patients at 40 to 60 years of age, no gender difference [6].

According to their origin, intrathoracic lipomas are classified into several types, such as endobronchial lipomas, diaphragmatic lipomas and this case belongs to pleural lipoma which originates from the submesothelial parietal pleura and may extend into subpleural, pleural or extrapleural spaces [6].

Lipomas can be also divided into two classes: (1) hourglass or dumbbell lipomas that pass through intercostal space or the thoracic inlet; and (2) purely intrathoracic lipomas. Our case belong to the latter type, since it was entirely within the right thorax [7,8].

Like our case, lipomas often grow slowly and subsequently are detected at a relatively late stage of evolution. But in Christophe’s opinion [2], such development was not so slow. Indeed, two of their patients with previously normal chest X-ray 7 months and 3 years ago respectively, presented with lipomas of 9 × 7 × 3 cm and 19 × 16 × 10 cm. therefore once lipoma is detected, close follow-ups are mandatory.

Most patients remain asymptomatic, but since lipomas are able to grow to a large size, they may provoke compression symptoms, which depend on sites and sizes of lipomas. Symptoms such as dyspnoea and dysphagia are show due to local compression on adjacent structures, such as the trachea or oesophagus. Our patient’s complaint of mild shortness of breath maybe due to the huge tumor’s compression on the right lung.

Jack et al. [9] reported a case of an intrathoracic, extrapericardial lipoma which was presented in a patient with severe left ventricular dysfunction. The patient refused surgical resection of the tumor and subsequently suffered cardiac arrest due to the tumor’s direct compression upon the heart.

Pleural lipomas also may cause complications such as intratumoral haemorrhage with pain and fever, moreover they can invade intercostal spaces and induce rib lysis [10].

Although the tumor is usually detected incidentally in a chest X-ray, CT scan has replaced conventional x-ray and ultrasound scan for accurate detection of thoracic lipomas. CT allows a definitive diagnosis when it demonstrates a homogeneous fat attenuation mass (-50 to -150 Hounsfield units, or HU) which formed obtuse angles with the chest wall and displaced adjacent pulmonary parenchyma and vessels [11]. The density may not be entirely uniform because lipomas often contain fibrous stroma. Ramona et al. [12] reported a case which CT revealed several areas of dystrophic ring-type calcifications within a field of scattered dense soft tissue elements.

The management strategy for pleural lipomas is still controversial. An observation principle with clinical and radiological follow-up may be suitable for elderly patients and those in poor general condition, especially in the presence of small and asymptomatic lesion [6].

But most authors argued surgical resection, if possible, would be recommended for diagnostic and therapeutic considerations.

In this case of large lipomas, surgical exeresis is then inevitable to diagnose and to treat prior to progression stage when complications may happen. It is also the reason why we did not performed fine-needle aspiration biopsy, which was such a less invasive method.

Surgical resection can easily be performed via muscle-sparing or an open typical thoracotomy, as in our case. Video assisted thoracoscopic surgery (VATS) has become a common technique for thoracic tumor which was pedunculated in form and small enough in size because there is no infiltrating growth in this type. It is an effective well-tolerated procedure that is associated with less morbidity and mortality. Recently, successful extirpation of a pleural lipoma with a single-port VATS has been reported [13].

The outcome of resection of lipomas is usually good. Recurrence rates after surgical excision have been reported to be less than 5% [6]. Most cases of recurrence are probably attributed to incomplete resection of the lesion. Factors such as infiltration of adjacent structures like the brachial plexus may impede complete resection of the lipoma and predispose to its recurrence.

Incomplete surgical resection might be performed rather than complete extirpative procedures which may cause a serious functional disorder. Wurlitzer and colleagues [14] reported several cases in which the tumor stopped growing after incomplete surgical tumor removal.

## Conclusion

Although pleural lipoma never evolves towards liposarcoma, surgical resection is still necessary due to the following reasons: firstly, preoperative diagnose is difficult to distinguish lipoma from well-differentiated liposarcoma; secondly, compression symptoms could be fatal; thirdly, complications such as intratumoral haemorrhage with pain and fever may exist during infiltrating development of the tumor preoperatively. During surgery, not only should we remove the tumor radically as far as possible, but we should also protect the surrounding tissues. After surgery, local recurrence of intrathoracic pleural lipoma is uncommon.

## Consent

Written informed consent was obtained from the patient for publication of this case report and accompanying images. A copy of the written consent is available for review by the Editor-in-Chief of this journal.

## Abbreviations

CT: Computed tomography; BMI: Body mass index; cm: centimeter; kg: kilogram; FVC: Forced vital capacity; FEV1: Forced expiratory volume in 1s.

## Competing interests

The authors declare that they have no competing interests.

## Authors’ contributions

MC and JY performed surgery. LZ carried out the patient diagnosis. MC and JY were major contributors in writing the manuscript. HZ Provide a lot of useful suggestions about this manuscript. All authors read and approved the final manuscript. Full list of author information is available at the end of the article.
